# Trombiculiasis in a Dog with Severe Neurologic Disorders, Spain

**DOI:** 10.3201/eid2604.191313

**Published:** 2020-04

**Authors:** Paula Santibáñez, Eva Gallo, Ana M. Palomar, Aránzazu Portillo, José A. Carrillo, José A. Oteo

**Affiliations:** Center of Rickettsiosis and Arthropod-Borne Diseases, Hospital Universitario San Pedro–Center for Biomedical Research from La Rioja (CIBIR), Logroño, Spain (P. Santibáñez, A.M. Palomar, A. Portillo, J.A. Oteo);; Vetersalud Asís Hospital, Logroño (E. Gallo, J.A. Carrillo)

**Keywords:** Trombiculidae, chiggers, canine trombiculiasis, neurological disorders, dog, dermatitis, zoonoses, bacteria, parasites, Spain, mites, Neotrombicula inopinata

## Abstract

Chiggers, the larvae of trombiculid mites, parasitize a wide variety of terrestrial vertebrates worldwide. Their bites cause seasonal trombiculiasis in humans and animals. Affected canines can have a variety of digestive and systemic clinical signs. We describe a case of canine trombiculiasis in a dog exhibiting severe neurologic symptoms.

Larvae of trombiculid mites (Acari: Trombiculidae), commonly known as chiggers, are widespread ectoparasites of vertebrates. More than 3,000 trombiculid species are known, and small mammals and birds are their main hosts (*1*). In Asia, chiggers transmit scrub typhus, a life-threatening human infection caused by *Orientia tsutsugamushi* (*2*). In Europe, chiggers are associated with a seasonal trombiculiasis, a dermatitis that affects humans and animals, mostly dogs and cats (*3*). Infected animals can have signs of skin lesions, pruritus, asthenia, fatigue, pyrexia, digestive disorders, or neurologic dysfunction (*3*–*5*). We describe a case of a dog in Spain heavily infested by chiggers and exhibiting severe neurologic symptoms.

On October 14, 2013, a 2-year-old male basset hound in poor health was admitted to Vetersalud Asís Hospital in Logroño, Spain. The owner reported that the dog had been vomiting and having trouble breathing for 12 hours before he was brought to the veterinary hospital. The dog was drooling, torpid and confused, vocalizing, and barely able to stand. His symptoms started the evening before with a lack of appetite, lethargy, and licking and biting his paws. The symptoms progressed to convulsions and ataxia so severe that the dog could no longer scratch itself. At initial evaluation, he had ataxia and loss of balance. Further clinical examination revealed an elevated body temperature of 39.6°C (normal 38°C–39°C), tachypnea with hyperventilation, and mucosal congestion. Skin examination revealed focal areas of erythema and papules on the abdomen, above the eyes, and in the interdigital areas. We performed skin scrapings on several lesions and needed tweezers to remove many mites that were attached to the dog’s skin (Figure, panel A). Microscopic examination of skin scrapings revealed numerous live mites, which we morphologically identified as *Neotrombicula inopinata* (*6*,*7*) (Figure, panel B). 

**Figure:**
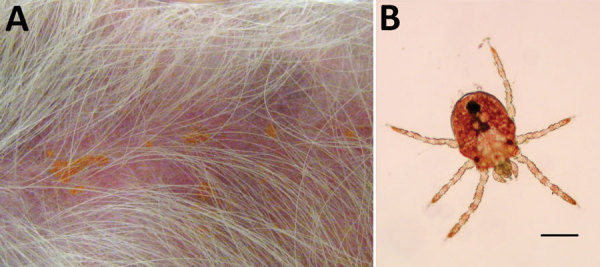
Larval *Neotrombicula inopinata* mites on a dog with severe neurologic symptoms, Spain. A) *N. inopinata* mites attached to the abdomen of the dog. B) Microscopic image of *N. inopinata* larva. Scale bar indicates 100 μm.

We treated the dog with phenylpyrazole, a broad-spectrum topical insecticide, and most of the mites detached within 12 hours. We treated the dog’s allergic reaction with methyl prednisolone at an initial dose of 10 mg/kg/day, which was gradually tapered to 0.5 mg/kg/day over 15 days. Complete blood count and chemical tests at admission, 24 h, and 48 h showed no changes except an increase in the number of neutrophils from 21.2 × 10^3^ cells/µL at admission to 35.4 × 10^3^ cells/µL at 48 h (upper limit 16.9 × 10^3^ cells/µL). The dog was hospitalized for 4 days, but ataxia was still evident after discharge (Video). Clinical signs went into remission 7 days after treatment began.

**Video:** A 2-year-old dog with severe neurologic symptoms caused by trombiculid mite infestation at discharge after 4 days in a veterinary hospital, Spain.

The dog had been walking and playing with its owners through grassy and brushy areas in a pine forest in Sierra Cebollera National Park, La Rioja, Spain (42°6′Ν, 2°33′Ε), ≈8 hours before the onset of symptoms. One owner had an itchy dermatitis that was diagnosed as trombiculiasis and treated at the Infectious Diseases Department at the Hospital Universitario San Pedro, Logroño, 4 days after the outdoor activities (*8*). We previously reported on trombiculid mites in the vegetation of certain areas of Sierra Cebollera National Park, their causality in human cases of seasonal dermatitis, and co-occurrence with canine cases (*4*,*6*,*8*–*10*). However, canine trombiculiasis associated with severe neurologic signs had not been described in Spain. 

In dogs, massive infestations with chiggers have been related to death, especially when left untreated (*10*). Orange spots, especially on the lacrimal areas, can assist in the diagnosis of suspected cases of trombiculiasis and should prompt owners to seek immediate veterinary advice. A severe allergic host response, hypersensitivity to mites or their products, or pathogen transmission have been speculated causes of clinical signs in canids (*3*). The role of *N. inopinata* mites collected in La Rioja as vectors of arthropodborne bacteria has not been demonstrated (*10*), and the clinical signs do not suggest an infectious disease process. We hypothesize that severe cases are attributed to the inflammatory response secondary to infestation, but the mechanism is unknown. However, we cannot disregard the implication of a neurotoxic process.

Successful management of symptoms is dependent on early treatment to remove chiggers. Topical insecticides, especially pyrethroids, are considered effective against chigger infestations in canids (*3*). Our experience has shown efficacy of isoxazolines, although not label indicated, at eliminating chiggers on dogs within 6–8 hours. As noted in this case, a short course of glucocorticoids at an antiinflammatory dose might be necessary to relieve pruritus and to reduce inflammation (*3*). Currently, no products are specifically licensed for preventing chigger bites. Sprays containing phenylpyrazole, which is licensed for use in dogs and cats against fleas and ticks, also are thought to be effective against mites. Because trombiculiasis is a seasonal threat, the most useful approach to prevent infestations, if feasible, consists of keeping pets away from areas where exposure can occur whenever chiggers are known to be active. Local veterinarians should be aware of the occurrence of canine trombiculiasis and its clinical signs to properly diagnose and manage this potentially fatal condition.
