# Supplementary figures and images for: Trombiculiasis in a Dog with Severe Neurologic Disorders, Spain
Source: Emerg Infect Dis. 2020 Apr;26(4):819–20. doi: 10.3201/eid2604.191313 (PMC7101131; doi:10.3201/eid2604.191313)

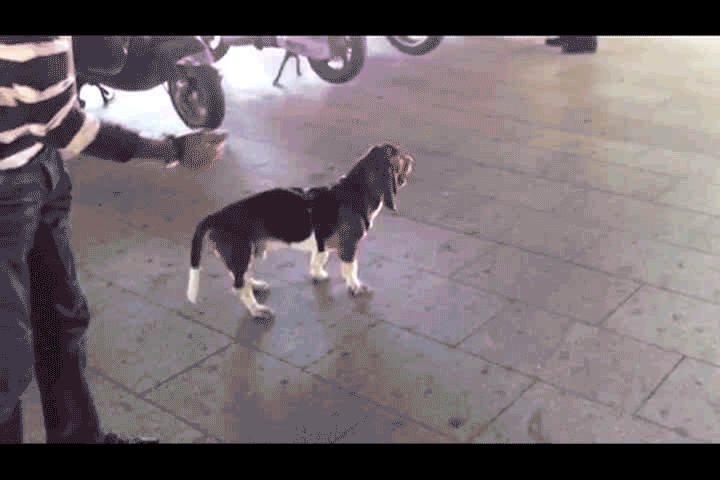

Supplement: Supplementary file 1 [file 19-1313-V.gif]
